# Removing motion and physiological artifacts from intrinsic BOLD fluctuations using short echo data

**DOI:** 10.1016/j.neuroimage.2012.09.043

**Published:** 2013-01-01

**Authors:** Molly G. Bright, Kevin Murphy

**Affiliations:** Cardiff University Brain Research Imaging Centre (CUBRIC), School of Psychology, Cardiff University, CF10 3AT Cardiff, UK

**Keywords:** fMRI, Intrinsic fluctuations, Resting state, Motion, Short echo time, Noise correction, Connectivity

## Abstract

Differing noise variance across study populations has been shown to cause artifactual group differences in functional connectivity measures. In this study, we investigate the use of short echo time functional MRI data to correct for these noise sources in blood oxygenation level dependent (BOLD)-weighted time series. A dual‐echo sequence was used to simultaneously acquire data at both a short (TE = 3.3 ms) and a BOLD-weighted (TE = 35 ms) echo time. This approach is effectively “free,” using dead-time in the pulse sequence to collect an additional echo without affecting overall scan time or temporal resolution. The proposed correction method uses voxelwise regression of the short TE data from the BOLD-weighted data to remove noise variance. In addition to a typical resting state scan, non-compliant behavior associated with patient groups was simulated via increased head motion or physiological fluctuations in 10 subjects. Short TE data showed significant correlations with the traditional motion-related and physiological noise regressors used in current connectivity analyses. Following traditional preprocessing, the extent of significant additional variance explained by the short TE data regressors was significantly correlated with the average head motion across the scan in the resting data (*r*^2^ = 0.93, *p* < 0.0001). The reduction in data variance following the inclusion of short TE regressors was also correlated with scan head motion (*r*^2^ = 0.48, *p* = 0.027). Task-related data were used to demonstrate the effects of the short TE correction on BOLD activation time series with known temporal structure; the size and strength of the activation were significantly decreased, but it is not clear whether this reflects BOLD contamination in the short TE data or correlated changes in blood volume. Finally, functional connectivity maps of the default mode network were constructed using a seed correlation approach. The effects of short TE correction and low-pass filtering on the resulting correlations maps were compared. Results suggest that short TE correction more accurately differentiates artifactual correlations from the correlations of interest in conditions of amplified noise.

## Highlights

► Short echo time fMRI data reflect head motion and physiological noise sources. ► Variance removed by short TE regressors correlates with total scan motion. ► Regressing short TE data from resting state BOLD data reduces artifactual correlations. ► This method could improve group comparisons of resting state data.

## Introduction

The utility of fMRI in investigating the workings of the brain is determined by our ability to capture and isolate appropriate signal contrast. Blood oxygenation level dependent (BOLD) contrast is the dominant method for identifying activated areas of the brain (Bandettini et al., 1992; Kwong et al., 1992; Ogawa et al., 1990). These signal changes reflect a complex coupling between local blood volume, blood flow and concentrations of deoxygenated hemoglobin, all of which are altered by neural activity (for a review, see Buxton, 2012). Typical fMRI experiments use fast imaging methods, such as gradient‐echo echo-planar imaging (EPI) to obtain data with high temporal resolution and large spatial coverage, with an echo time (TE) optimized for BOLD contrast. In addition to mapping task-related activity, these techniques have been applied to mapping intrinsic or “resting state” BOLD fluctuations that occur in the absence of a task (Biswal et al., 1995; Biswal, 2012). These fluctuations have demonstrated key networks in the brain whose connection strengths may be altered during aging and disease (Broyd et al., 2009).

Functional connectivity networks are typically mapped using seed correlation techniques; in particular, the default-mode network (Greicius et al., 2003) is readily extracted using a seed region in the posterior cingulate cortex. Using this approach it has been shown that the connectivity within this network may be altered in pathology (see Broyd et al., 2009; Snyder and Raichle, 2012). However, recent publications have called into question studies comparing connectivity across groups, showing previous conclusions may be erroneous when motion artifacts are more accurately dealt with in data processing (Power et al., 2012; Van Dijk et al., 2012). This is particularly problematic when comparing children or patient populations with young healthy adults as these groups are likely to be more uncomfortable in the MR environment and thus may move differently. Also, these studies suggest that subtle motion artifacts, rather than large head movements alone, can confound connectivity analyses.

Unfortunately, isolating the true signal changes of interest is an ongoing challenge. Over the years, much focus has been placed on removing various sources of noise from BOLD data. In addition to thermal noise, scanner drift, distortions and signal drop-out related to field inhomogeneities, the volunteer also contributes several noise sources to the data. These noise sources not only result from gross motion artifacts but can also be related to physiological rhythms such as cardiac and respiration cycles. For example, the act of respiration causes multiple types of noise: breathing may cause the subject's head to move slightly during the scan, thereby altering spin history in a spatially dependent manner (Friston et al., 1996), the movement of the subject's chest can alter the magnetic field in a time-dependent manner (Brosch et al., 2002), and fluctuations in breathing can alter arterial blood gas tensions and thus cerebral blood volume and blood flow (Birn et al., 2006; Wise et al., 2004). Many noise sources can be addressed during the acquisition stage: padding and restraints can be used to limit head motion, real-time shimming can be incorporated to reduce the effects of chest motion on the main magnetic field (van Gelderen et al., 2007), and end-tidal forcing systems can be incorporated to maintain stable breathing rates and arterial blood gas tensions (Wise et al., 2007). Alternatively, several analysis steps have been developed to measure, model, and retrospectively remove the effects of various noise sources from the BOLD-weighted data set (e.g., motion correction/modeling, RETROICOR and RVHRcor; Chang et al., 2009; Glover et al., 2000; Jenkinson et al., 2002).

Another approach is to use data acquisition strategies that differentiate BOLD signal changes of interest from nuisance sources of signal variance. Using multiple echo acquisitions, changes in the local relaxation rate *R*_2_* (which is the mechanism underlying the BOLD contrast) can be isolated. Beginning in the late 1990s, several groups began to explore the use of this technique in differentiating changes in initial signal intensity (*I*_0_ or *S*_0_) from changes in relaxation rates, in particular *R*_2_* (Barth et al., 1999; Posse et al., 1999; Speck and Hennig, 1998). A review of the resulting rush of developments in multi-echo techniques is presented by Posse (2012). In the early studies, the short TE data were identified as possible noise correction tools, being able to distinguish inflow effects from BOLD activation (Glover et al., 1996; Speck and Hennig, 1998). The effects of gross head motion on functional fMRI data have also been addressed by using short TE data to de-noise BOLD-weighted data confounded by large motion artifacts (Buur et al., 2009).

Physiological noise, typically removed by using noise regressors based on externally measured data (e.g., pulse, respiration, etc. (Birn et al., 2006; Chang and Glover, 2009; Glover et al., 2000)) may also be represented in short TE data due to the related pulsations or fluctuations in blood volume and inflowing spins with altered saturation. As with motion, it is plausible that the short TE data may contain additional information regarding the local physiological variations, beyond that explained in the empirically derived models.

Improved ability to remove physiological and motion artifacts from BOLD-weighted data may help address the challenges faced when comparing distinct populations. This is extremely important when interpreting “functional connectivity” differences between subject groups that are likely to exhibit different noise characteristics. Altered noise structure leads to changes in correlation between region-specific BOLD time series (Lund et al., 2006). Such changes are normally attributed to altered “connectivity,” suggesting that the neuronal activity underpinning the BOLD signal has changed. As both the Van Dijk and Power papers have shown, noise structure differences between groups caused by something as simple as the amount of movement during the scan can drastically alter the interpretation of a study and lead to erroneous results. Similar problems may be caused by physiological differences between groups such as systematic changes in respiration rate (e.g. one group may be more nervous than another) (Birn et al., 2008b).

In this study, we investigate the use of simultaneously collected short echo time data to correct for spurious noise sources in BOLD‐weighted time series. The factors that contribute to fMRI signal variance at short TE are determined, focusing on motion and physiological noise, which are two confounds commonly seen in patient studies. Task-related data are used to demonstrate the effects of short TE correction when the BOLD neural activity signature is known. Gross motion and physiological artifacts are introduced into “resting state” data to simulate a non-compliant patient population. The short TE data are used to correct for these confounds in the BOLD-weighted data, and the effects on the intrinsic fluctuation correlation patterns in individual subject and group data sets are explored.

## Methods

### Data acquisition

Ten subjects were scanned using a 3‐T GE HDx scanner (Milwaukee, WI, USA) equipped with an 8-channel receive head coil. Data were acquired using a dual-echo gradient echo spiral readout sequence (TR = 2000 ms; 64 spirals; FOV = 22 cm; 18 slices; resolution = 3.4 × 3.4 × 5.0 mm^3^). The first echo time (TE1), also referred to as the “short TE,” was set to be 3.3 ms in the majority of scans (sometimes set to 10 ms, see below). The second echo time (TE2) was always defined as 35 ms to provide typical BOLD-weighting.

Subjects were presented with cues and stimuli using a rear projector and screen viewed through a mirror on the head coil. Six functional dual-echo scans were acquired.

In the *Rest* scan, the subject was asked to remain at rest with their eyes open while a steady fixation cross was displayed in the center of the screen. In the Rest + Motion scan, the fixation cross briefly changed color at approximately 10 s (jittered) intervals, which cued the subject to nod their head in a random direction of their choosing. In the Breathing scan, the subject was prompted to perform 3 Breath Hold (BH) and 3 Cued Deep Breathing (CDB) respiratory challenges via text-based instructions projected onto the screen (as described in Bright et al., 2011). In the Visual_3.3_ scan, a TE1 of 3.3 ms was used and a block design flashing checkerboard stimulus was presented (8 Hz, 30 s blocks, full contrast). In eight subjects, this scan was repeated with TE1 = 10 ms; this scan is referred to as Visual_10_. In all subjects, the Visual_3.3_ scan was also repeated with the additional cued head motion as described above, and this scan is referred to as Visual_3.3_ + Motion. All scans contained 165 repetitions (5.5 min), except the Breathing scan (244 repetitions, 8 min 8 s).

A whole-brain low-resolution image was acquired using the same dual-echo spiral sequence, in addition to a whole-brain high-resolution *T*_1_-weighted structural image (resolution = 1 × 1 × 1 mm^3^), for the purposes of image registration.

Subjects were equipped with multiple physiological monitoring devices: respiratory bellows, a pulse oximeter, and a nasal cannula connected to CO_2_ and O_2_ gas analyzers (AEI Technologies, PA, USA) were used to continuously record physiological data during all scans.

### Data preprocessing

Data were preprocessed in two ways: traditional preprocessing and traditional preprocessing with additional TE1 data regressors.

#### Traditional data preprocessing

Although there is not yet a standard method for preprocessing resting state, activation, and respiratory challenge fMRI data, the majority of correction factors widely accepted in the field (Birn, 2012) were applied to the BOLD-weighted TE2 data in this study.

First, we performed motion correction on the TE2 data (MCFLIRT, FSL, Oxford, UK; Jenkinson et al., 2002), and the resulting transformations were applied to the corresponding TE1 data. This has been shown to be an appropriate method for correcting dual-echo data sets to maintain co-registration (Jonsson et al., 1999). The first three volumes were removed from each scan, non-brain matter was removed (BET, FSL, Oxford, UK), and quadratic detrending was performed (AFNI, NIH, Bethesda, MD, USA). Next, 25 noise regressors were removed from the TE2 data:

- Six motion transformation time series (*x*-, *y*-, *z*-translations, pitch, roll, yaw) that were obtained during the motion correction analysis,
- The derivatives of each of the six motion transformation time series,
- Eight RETROICOR regressors (four respiratory and four cardiac) that were calculated from the respiratory bellows and pulse oximeter data (Glover et al., 2000),
- End-tidal oxygen (P_ET_O_2_) and end-tidal carbon dioxide (P_ET_CO_2_) values that were automatically extracted from the O_2_ and CO_2_ traces obtained from the gas analyzers and interpolated to the acquisition timepoints. These traces were convolved with a standard hemodynamic response function (Murphy et al., 2011),
- The respiration volume per time (RVT) regressor was calculated from the respiratory bellows data and convolved with a respiratory response function (RRF) (Birn et al., 2008a),
- The cardiac rate was calculated from the pulse oximeter data and convolved with the cardiac response function (Chang et al., 2009; Shmueli et al., 2007),
- A CSF regressor, created by averaging the voxel time series within an eroded mask of the lateral ventricles.

Thus noise regressors representing gross head motion, physiology-related motion (e.g., pulsation), and changes in blood volume/flow related to arterial gas tensions were regressed out of the TE2 data. The global signal was not regressed from the data due to possible confounds this procedure can introduce (Murphy et al., 2009).

#### TE1 regression

Working under the hypothesis that the short TE data contains additional information regarding noise sources, we incorporated the TE1 data as voxelwise noise regressors in addition to all 25 regressors used during the traditional preprocessing pipeline described above (AFNI, NIH, Bethesda, MD, USA).

### Data analysis

#### Characterization of short echo variance

In order to compare the noise information contained in the short TE data and the externally measured/calculated noise regressors, correlation coefficients between each of the 25 noise regressors described above and the TE1 data of each scan were calculated.

The average scan motion was calculated as the mean frame-wise displacement (FWD) using the method described by Power et al. (2012). The results of the motion correction (FLIRT, FSL) were used to measure the relative change in the three translation (*x*, *y*, and *z*) and rotation parameters (pitch, roll, and yaw) between each scan volume *n* and the preceding volume *n* − *1*. The rotational displacements were converted from degrees to millimeters by calculating the corresponding displacement on the surface of a sphere with a 50-mm radius (approximately the mean distance from the cerebral cortex to the center of the head; notated as *a*, *b*, and *c*). For each scan volume *n*, the FWD was defined as

$$\text{FWD}=\sqrt{{\left(x_{n}-x_{n-1}\right)}^{2}+{\left(y_{n}-y_{n-1}\right)}^{2}+{\left(z_{n}-z_{n-1}\right)}^{2}+{\left(a_{n}-a_{n-1}\right)}^{2}+{\left(b_{n}-b_{n-1}\right)}^{2}+{\left(c_{n}-c_{n-1}\right)}^{2}}$$

and this parameter was averaged across the scan to provide an estimate of total motion.

Voxels in which the short TE regressors explained significant additional variance were identified using an *F*-test; the number of these voxels were calculated and compared with the mean FWD of the scan.

Data variance was also quantified using the DVARS metric which has been related to head motion (described in Power et al., 2012), following traditional preprocessing and following additional short TE correction. DVARS is calculated as the root-mean-square of the derivatives of the timecourses of all brain voxels for each volume, thus providing a measure of the change in intensity from one volume to the next. The correlation between the DVARS and FWD traces was calculated for all data sets and processing methods. Finally. the percentage change in average scan DVARS associated with the addition of short TE regressors was compared to the average scan FWD to determine whether the variance removed by short TE correction relates to head motion.

#### Short TE data correction in task-related BOLD data

As we are not able to acquire data at TE = 0 ms, it is possible that there is also some BOLD-weighting in the signal variance of the TE1 data set. To test the influence of this BOLD contamination on the use of TE1 data as a noise correction tool, the TE2 activation maps of the Visual_3.3_ and Visual_10_ and Visual_3.3_ + Motion data sets after traditional preprocessing and after the extended TE1 regression preprocessing were obtained and compared using AFNI. An ROI of the occipital lobe defined in MNI space (MNI152, nonlinearly derived, McConnell Brain Imaging Centre, Montreal Neurological Institute, McGill University, Montreal, Canada; Mazziotta et al., 2001) was transformed into the functional data space and the number of significantly activated voxels in this ROI (*p* < 0.05, Bonferroni corrected) and the mean *t*-statistic of these voxels were compared between the different TE1 values and preprocessing pipelines.

#### Short TE data correction in “resting state” seed correlation analysis

As discussed previously, seed correlation maps are one possible and important application for improved noise correction methods. Seed correlation maps of the default mode network (DMN) of individual subjects and the group average correlation map were calculated for the Rest, Rest + Motion, and Breathing data sets to determine the benefits of the TE1 denoising in the presence (or absence) of large motion artifacts or physiological variance. The functional data were smoothed with a Gaussian kernel (FWHM = 5 mm). A seed region in the posterior cingulate cortex (PCC) was defined by drawing a 12-mm‐diameter sphere centered around the previously published Talairach coordinate [5L, 49P, 40S] (Fox et al., 2005; Murphy et al., 2009; Shulman et al., 1997). The time series of voxels within the seed region were averaged together, and the correlation between this seed time series and every voxel in the brain was calculated.

The functional data sets and correlation maps were aligned to the appropriate structural image, which in turn was aligned to MNI space (FLIRT, FSL, Oxford, UK; Jenkinson et al., 2002), transformed into z-scores using Fisher's r-z transform, and the individual subject as well as group average correlation maps were thresholded for significance (corrected cluster significance *p* < 0.005).

The Van Dijk et al. (2012) study of resting state correlations in 1000 subjects showed that, although motion was not responsible for the majority of their observed correlations, motion was responsible for decreasing long-distance correlation and increasing local correlations. Thus, we also assessed the effect of TE1 regression on local and long-distance correlation in the Rest, Rest + Motion, and Breathing data sets. A long-distance ROI was defined as the significant cluster in the medial-frontal cortex of the group correlation map of the Rest data set following traditional preprocessing. The PCC ROI was extended to a sphere with a diameter of 36 mm to create a “local” ROI. The mean *z*(*r*) statistic was calculated in each data set of each subject in the local and long-distance ROIs before and after TE1 regression.

Finally, to better understand our proposed short TE analysis method in the context of existing methodology, we compared the changes in *z*(*r*) associated with short TE correction with the changes related to low-pass filtering of the data. Following the traditional preprocessing steps, an additional low‐pass filter (< 0.1 Hz) was applied with the aim of removing high-frequency artifactual noise, and PCC seed correlation analysis was performed. Significant differences in the *z*(*r*) maps following low-pass filtering or following short TE correction were determined (paired *t*-test, corrected cluster threshold *p* < 0.05) and compared in the Rest, Rest + Motion, and Breathing data sets.

## Results

### Noise sources contributing to short echo variance

Significant correlations were observed between voxelwise timecourses of the TE1 data and the traditional noise regressors, although the strength and extent of correlations varied greatly across subjects and scans. A full list of the number of significantly correlated voxels for each subject's Rest, Rest + Motion, and Breathing scans and all noise regressors is provided in the supplementary material (Table S1). Relative to the Rest scan results, the Breathing data set showed significantly more voxels correlated with the RVT regressors, while the Rest + Motion data set showed fewer voxels correlated with certain RETROICOR regressors (*p* < 0.05, paired *t*-test, corrected for multiple comparisons). This shows that more physiological noise is present in the short TE data of the Breathing scan, related to the respiratory challenges, but less physiological noise is observed in the Rest + Motion data, where gross head movement artifacts may overwhelm more subtle pulsatory motion. There is no significant increase in the representation of the individual motion regressors in the short TE data of the Rest + Motion scan relative to the Rest scan across subjects, which likely reflects the inter-subject variation in head movements during the motion stimulus. The total scan motion in the Rest, Rest + Motion, and Breathing data for all subjects is presented in Table 1.

**Fig. 1:**
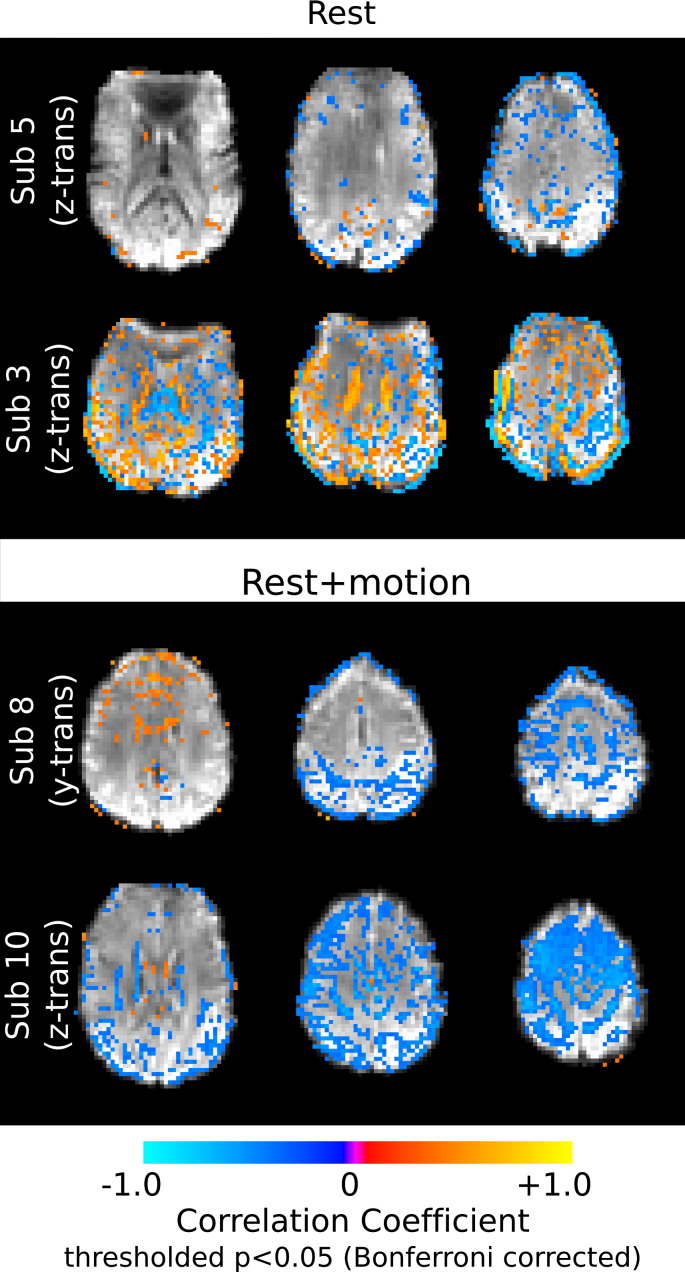
Significant correlation of short TE data (TE = 3.3 ms) and selected motion regressors (thresholded at *p* < 0.05, Bonferroni corrected). Correlation maps are presented for subjects exhibiting the median (top rows) and maximum (bottom rows) total scan motion in the Rest and Rest + Motion data sets (maps illustrate significant correlations with the motion regressor exhibiting the greatest number of correlated voxels in that subject's data (as determined using the results provided in the supplementary material). Not shown are the correlation maps of subjects exhibiting the minimum number of correlated voxels, as there were subjects with zero significant correlations with at least one motion regressor in both data sets.

**Fig. 2:**
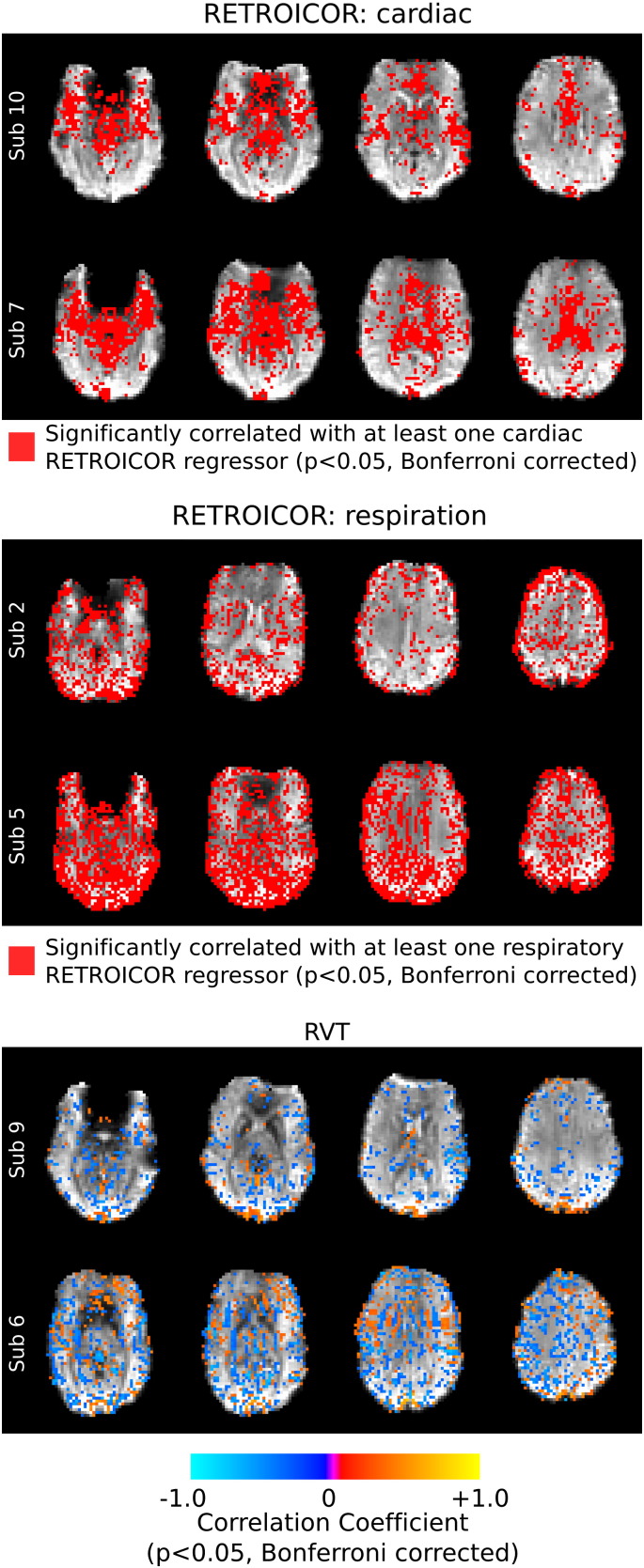
Significant correlation of short TE data (TE = 3.3 ms) and physiological regressors (*p* < 0.05, Bonferroni corrected). Binary maps of voxels where the short TE time series were significantly correlated (or anticorrelated) with any of the four cardiac or four respiratory RETROICOR regressors are shown, as well as thresholded correlation maps of the short TE data and the RVT regressor. The subjects presented exhibited the median (top rows) and maximum (bottom rows) numbers of significantly correlated voxels, as discussed in the supplementary material.

To illustrate typical spatial distributions of these correlations, maps of selected subjects are provided in Figs. 1 and 2. The subjects with the median and maximum total scan motion in the Rest and Rest + Motion data sets were identified (Table 1). Fig. 1 shows voxels where the short TE data were significantly correlated with the motion regressor that exhibits the greatest number of correlated voxels in that subject's data (*p* < 0.05, Bonferroni corrected). This demonstrates that the short TE data reflect motion artifacts even in true “resting state” data without additional cued head movements. Fig. 2 shows binary maps of voxels significantly correlated (or anticorrelated) with any of the four cardiac or four respiratory RETROICOR regressors and the correlation maps associated with the RVT regressors; subjects were selected as those with the median and maximum number of correlated voxels, summed across the four cardiac, four respiratory regressors, or the single RVT regressor, respectively. These maps indicate widespread correlations between the short TE data and multiple physiological noise sources.

### Additional BOLD data variance explained by short TE regressors

A voxelwise *F*-test identified voxels in which the inclusion of the short TE regressor explained significant additional variance in the BOLD-weighted data set beyond that which could be explained using the traditional noise regressors alone (*p* < 0.05, Bonferroni corrected). In the Rest, Rest + Motion and Breathing data sets, the short TE regressors explained more variance than traditional preprocessing in 26.48%, 68.05% and 27.06% of brain voxels respectively (see Table 2). Across the group, the number and the mean *F*-statistic of these voxels were significantly greater in the Rest + Motion data set than in the Rest data set. This demonstrates that with larger motion, more variance is explained in significantly more voxels by the inclusion of short TE regressors. In contrast, there was no significant difference in the number of voxels or mean F-statistic in the Breathing and Rest data sets across the group. This indicates that the short TE data do not capture additional physiological variance beyond that explained by traditional preprocessing. However, the short TE regressors explained additional variance in approximately 26% of brain voxels in both of these data sets, presumably related to motion confound removal.

**Fig. 3:**
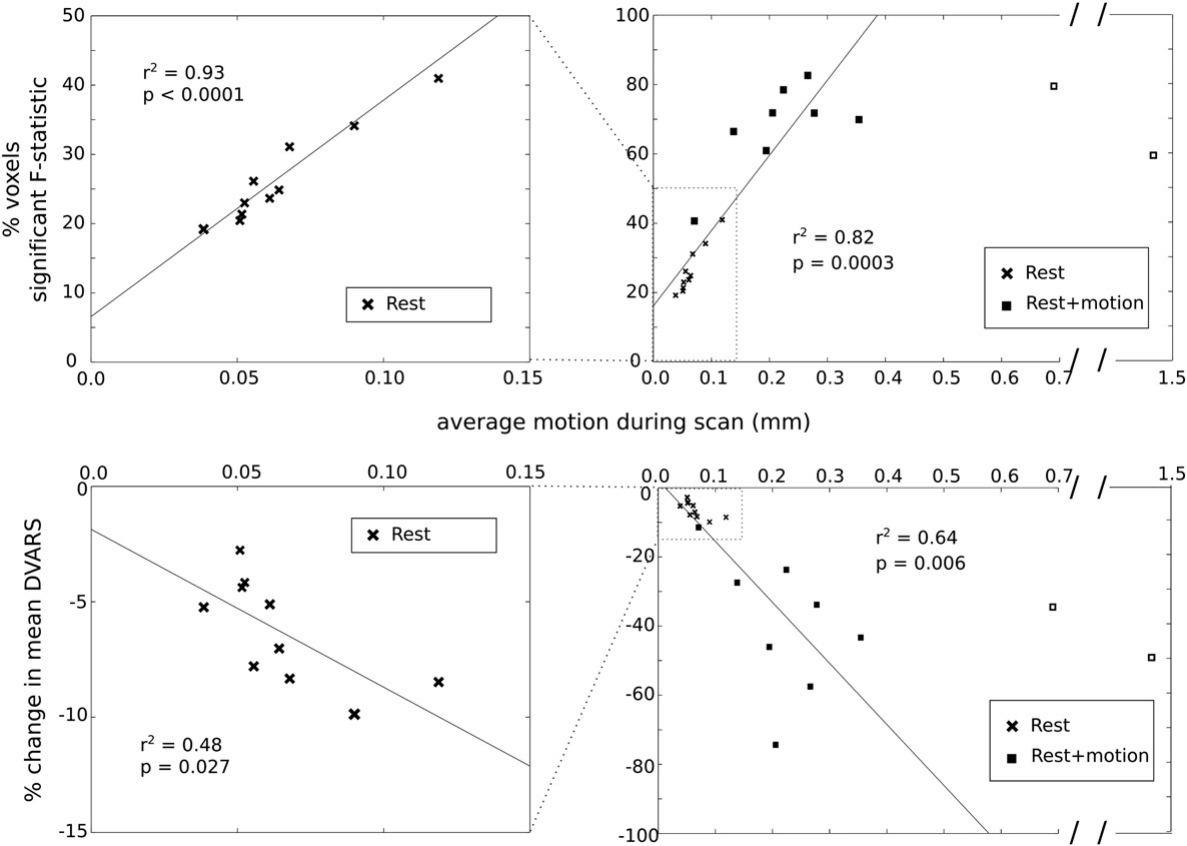
Top row: the percentage of brain voxels where short TE data regressors explain significant additional variance is strongly correlated with the average head motion across the scan (*r*^2^ = 0.93, *p* < 0.0001) in the Rest data set. This correlation continues when the head motion is amplified (combined Rest and Rest + Motion data, *r*^2^ = 0.82, *p* = 0.0003). Note that the Rest + Motion data of two subjects were identified as outliers, with average scan head motion more than three standard deviations from the combined group mean, and excluded from the correlation analysis. These datapoints are identified as open squares. Bottom row: the percentage reduction in data variance (average scan DVARS) following short TE data correction is strongly correlated with the average head motion across the scan in the Rest (*r*^2^ = 0.48, *p* = 0.027) and in the combined Rest and Rest + Motion data, excluding the same two outliers as above (*r*^2^ = 0.64, *p* = 0.006).

In support of this hypothesis, the percentage of voxels in which the short TE regressor explained significant additional variance was strongly correlated with the mean FWD in the Rest data set (*r*^2^ = 0.93, *p* < 0.0001). The Rest + Motion data followed the same trend as the Rest data, following the removal of two outlier data sets (FWD more than three standard deviations from the mean): the combined data also exhibited strong correlation (*r*^2^ = 0.82, *p* = 0.0003). These results are illustrated in Fig. 3. This again demonstrates that with greater amounts of motion, the short TE regressors explain greater amounts of variance.

The correlation between the FWD and DVARS traces in the Rest data set exhibited great inter-subject variability (correlation coefficients ranging from − 0.06 to 0.72 following traditional preprocessing). This correlation was significantly reduced following the inclusion of short TE correction (group average *z*(*r*) = 0.32 to 0.30, *p* = 0.02, paired *t*-test), which indicates that data variance and head motion become less correlated during this correction step. The average scan FWD and the percentage change in average scan DVARS following the addition of short TE correction were significantly correlated in the *Rest* data set (*r*^2^ = 0.48, *p* = 0.027) and in the combined Rest and Rest + Motion data, excluding the same two outliers as above (*r*^2^ = 0.64, *p* = 0.006). These results are displayed in Fig. 3, and provide more evidence that the data variance removed by short TE correction is associated with the amount of head motion in the data across difference scales of motion artifact.

### Removing short echo variance from task-related BOLD data

Our ability to use the TE1 data to remove additional noise variance from the simultaneously acquired TE2 data is directly challenged by the presence of BOLD contamination in the TE1 data set. Table 3 describes the number of activated voxels in the occipital cortex and the significance of the activation with and without the addition of short TE data regressors in the Visual_3.3_ and Visual_10_ and Visual_3.3_ + Motion data sets in eight subjects (two subjects did not complete this scan and were not available for a second session). When TE1 = 3.3 ms, in the absence of cued head movements (Visual_3.3_ data), the short TE regressors reduce the size of activation by 16.5% and the mean *t*-statistic of activation by 15.2% (averaged across subjects). When TE1 = 10 ms, this effect is exacerbated and activation is reduced by approximately 32-34%. In contrast, with increased head motion artifacts in the Visual_3.3_ + Motion data set, the strength and mean *t*-statistic of activation are *increased* by 23.2% and 41.9%, respectively. This indicates that, although the short TE regression can increase significance of activations when there are large amounts of motion, BOLD contamination or correlated blood volume effects in the short TE data can also reduce the statistics. This effect is mitigated (but not fully resolved) when the TE is reduced from 10 ms to the shorter 3.3 ms.

### “Resting State” seed correlation analysis

**Fig. 4:**
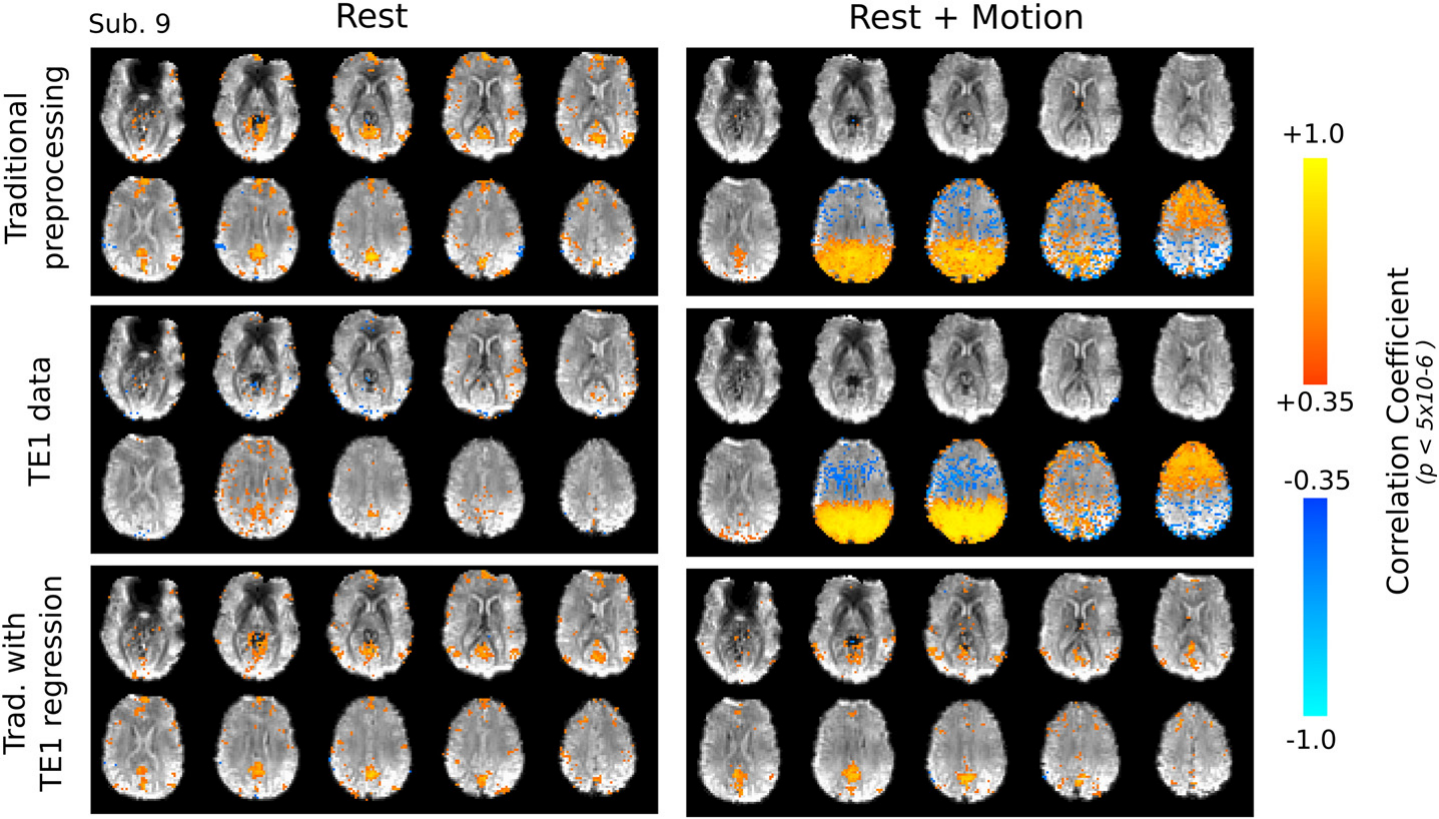
Unsmoothed seed correlation maps of one subject (thresholded at *p* < 5 × 10^− 6^). The Rest data exhibit the expected default mode network following traditional preprocessing, and the additional regression of TE1 data maintains the qualitative network pattern. In the Rest + Motion data of this subject, traditional preprocessing of the BOLD-weighted TE2 data does not resolve the default mode network; instead, the correlation map is dominated by gross head motion artifacts. These artifacts are also present in the TE1 Rest + Motion data set, and the TE1 regression method better resolves the expected network map in the TE2 data.

Fig. 4 compares the seed correlation maps of the DMN in the Rest and Rest + Motion data sets for one subject (unsmoothed correlation coefficients, thresholded at *p* < 5 × 10^− 6^). This subject represents our “best case” example, and the correlation maps qualitatively demonstrate the potential benefits of the short TE data correction technique (the quantitative group results are presented later). In the Rest data, the traditional preprocessing results in the expected DMN pattern, and including the short TE data regression maintains the qualitative structure of this network map. In contrast, seed correlation in the Rest + Motion data set does not result in the expected DMN following traditional preprocessing. Instead, the maps are dominated by the gross head motion artifacts caused by the cued nodding task. Seed correlation mapping in the corresponding TE1 data demonstrates the same artifacts, and the TE1 regression removes some of this variance from the BOLD-weighted TE2 data set, recovering much of the underlying network.

**Fig. 5:**
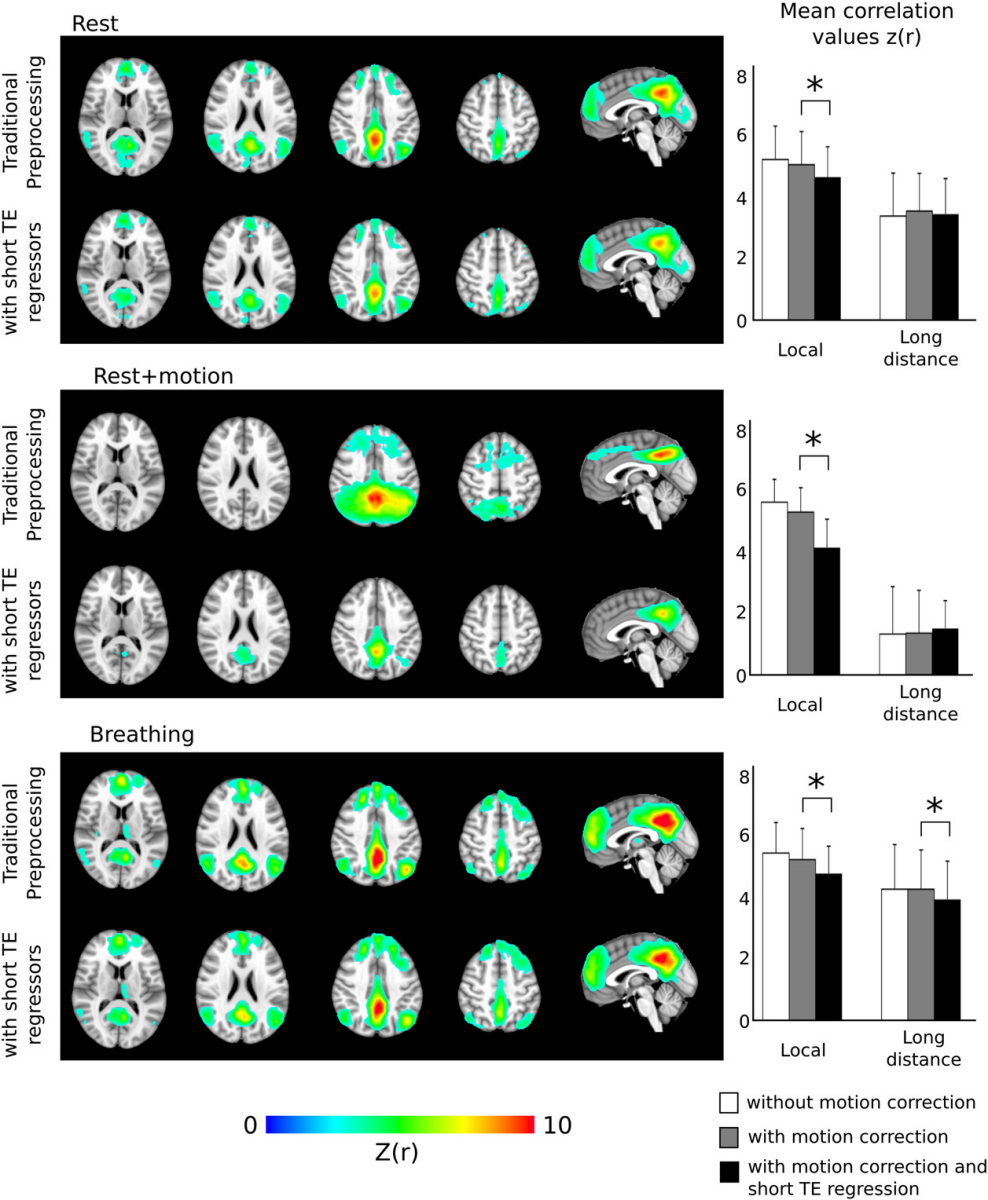
Group average seed correlation maps of the default mode network of 10 subjects (cluster threshold *p* < 0.005) in the Rest, Rest + Motion, and Breathing data sets following traditional preprocessing and with the addition of short TE data regressors. The mean *z*(*r*) statistics within local and long-distance ROIs were calculated in each data set of each subject, and the group averages are presented graphically. The local correlation values were significantly reduced in all data sets (paired *t*-test, *p* < 0.05 corrected for multiple comparisons) and the long-distance correlation values were significantly reduced in the Breathing data only.

The group average seed correlation maps for the Rest, Rest + Motion, and Breathing data across the 10 subjects in our study are presented, in MNI-space, in Fig. 5 (*z*(*r*) statistics; cluster threshold *p* < 0.005).

Fig. 5 also presents the changes in local and long-distance correlations after applying the TE1 data regressors in addition to traditional preprocessing methods. The mean *z*(*r*) of “local” correlations within the extended seed region is significantly decreased (*p* < 0.05, corrected for multiple comparisons) in all data sets, while the “long-distance” correlations were significantly reduced in the Breathing data set only.

**Fig. 6:**
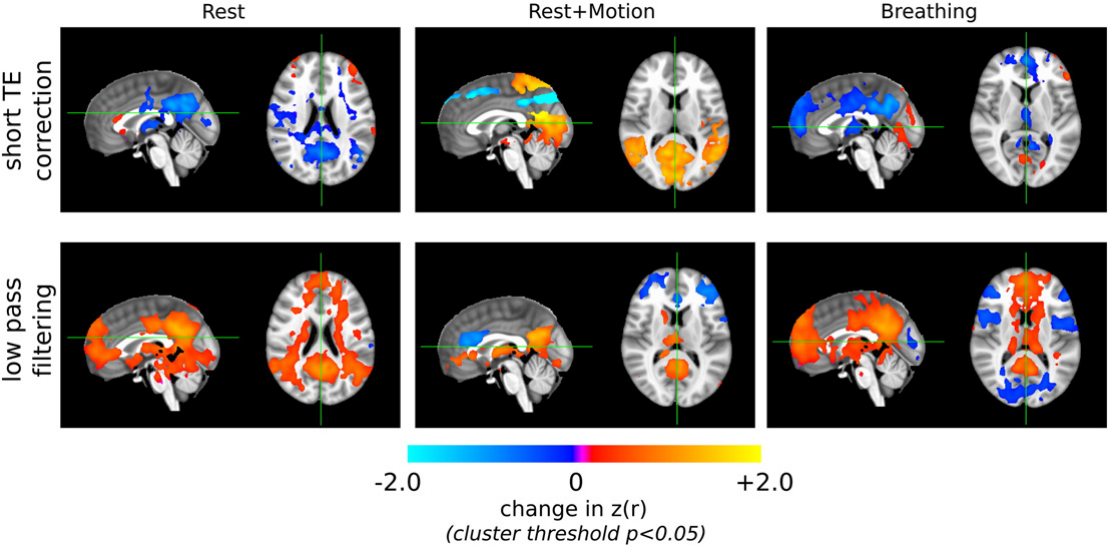
Significant changes in correlation values following short TE correction or following low-pass (< 0.1 Hz) filtering (corrected cluster threshold *p* < 0.05). The short TE correction significantly reduced DMN correlation values in the Rest data, in agreement with Fig. 5, whereas low-pass filtering increased correlation values. In the Rest + Motion data, short TE correction resulted in greater enhancement of DMN correlation values than low-pass filtering, simultaneously reducing other correlations associated with large head motion. In the Breathing data, low-pass filtering increased artifactual correlations near ventricles, likely related to uncorrected physiological noise, whereas short TE correction reduced these effects.

The significant changes in correlations after applying TE1 data regressors and after applying low-pass filtering (< 0.1 Hz) are compared in Fig. 6. As expected, by removing a great deal of data variance, the low-pass filtering greatly amplifies correlation values in the Rest data, whereas the short TE correction method is more subtle and mostly results in decreased correlation values (in agreement with the Fig. 5). Conversely, in the Rest + Motion data, the short TE correction method results in greater increases in *z*(*r*) in the DMN regions, while the low-pass filtering method is mostly limited to *z*(*r*) increases in the PCC (seed region). In the Breathing data, the two methods have very different effects on *z*(*r*); of note, correlation values near the ventricles are enhanced after low-pass filtering and reduced following short TE correction.

## Discussion

In this study, we have mapped the correlation between short TE functional data and traditional noise regressors reflecting head motion and physiological fluctuations, both of which may be amplified in patient groups.

### Motion

Recently, a study of resting state correlations in 1000 subjects (Van Dijk et al., 2012) showed that, although motion was not responsible for the majority of observed correlations, motion was responsible for decreasing long-distance correlation and increasing local correlations. This phenomenon is demonstrated in the data presented in the current study, where the Rest + Motion seed correlation maps following traditional preprocessing show excessive correlation values across the posterior cortices near the PCC seed region (e.g., Fig. 4).

In addition, the study by Van Dijk et al. compared groups of subjects with differing amounts of motion and observed significant artificial differences in “connectivity.” A similar study showed that motion artifacts cause spurious correlations in resting data, even after motion correction methods and the regression of the motion transformations (Power et al., 2012). These two papers have extreme consequences in the field of fMRI connectivity: an earlier paper showing a difference in connectivity reflecting brain maturation and development is now considered to reflect motion artifacts rather than true neuronal differences (Power et al., 2012). The application of short TE data for correcting gross head motion artifacts has been recently presented by Buur et al. (2009). In that study, data were acquired at TE1 = 14 ms (at 1.5 T) or TE1 = 9 ms (at 3 T) and the scan focused on continuous head nodding or self initiated motion during blocks of visual stimulation.

In the current study, we bring these studies together: we examine typical resting state data and also utilize a sporadic random direction motion stimulus to simulate extreme non-compliant patient behavior. We then assess the relationship between the benefits of short TE data regression and total scan motion and the effect of TE1 data correction on the resulting seed correlation maps. As even *subtle* differences in head motion have been shown to influence group connectivity results, it is important to assess short TE correction methods across a broad spectrum of head motion artifacts by examining the Rest data set in addition to the Rest + Motion data set. The strong correlation between the explanatory power of the short TE regressors and the mean FWD (Fig. 3) indicates that the short TE data correction method is closely related to motion artifacts in scans with both subtle and extreme head movements. Short TE data regression could thus be applied to reduce the confounding effects of motion in BOLD fMRI resting state data, in individual subjects and in group analyses, in situations with both small and large head motion artifacts. This type of technique would also avoid the possible negative consequences of deleting or “scrubbing” motion-contaminated timepoints recently proposed (Power et al., 2012). Although modifying the original scrubbing procedure can prevent motion artifacts from extending to adjacent volumes, which would therefore also need to be excluded (Carp, in press), scrubbing invariably removes data from analysis, reducing statistical power. More subtle motion artifacts, often in lower frequencies, would be invisible to a threshold-based scrubbing technique; in contrast, we have shown that the subtle head motions in the Rest data are at least partially corrected for using short TE correction.

### Physiology

Our study produces less conclusive results regarding the use of short TE data for physiological noise correction. We show extensive correlation between physiological noise regressors and the TE1 data (e.g., Fig. 2), however the short TE regressors explain similar amounts of significant additional variance in the Breathing data as in the Rest data. This suggests that the traditional noise regressors currently employed by the fMRI field (i.e., end-tidal gas levels, RVT, cardiac rate) are sufficient for denoising data with large physiological noise artifacts, and the short TE regressors do not explain any further physiology-related variance. This is probably due to the fact that they are global noise signals producing temporally similar but varying amplitude noise fluctuations in each voxel. There may be some beneficial noise correction that does not reach significance across the *F*-test group analysis; the long-distance correlations in the Breathing data are significantly reduced following short TE data regression (Fig. 5), suggesting that additional widespread physiological noise is being removed.

However, there may be alternative uses for short TE data for quantifying, rather than correcting for, physiological fluctuations. This is discussed in more detail below.

### Short TE correction versus low-pass filtering

Low-pass filtering is a common technique for reducing the confounds of higher-frequency noise sources such as unaliased respiration and cardiac pulsations, or fast head motion artifacts. Many of these noise sources are also present in the TE1 data presented in this study (Figs. 1 and 2). However, our results suggest that low-pass filtering and short TE correction can impact seed correlation maps in very distinct ways.

In the Rest data set, it is unsurprising that low-pass filtering results in significantly greater correlation values throughout the DMN. In simulated time series of the same length and temporal resolution as the fMRI data in this study, and with a temporal signal-to-noise ratio of 80 (based on Murphy et al., 2007), a < 0.1 Hz low-pass filter reduced data variance by approximately 34%. In addition, approximately 96 of 160 degrees of freedom are also lost by this filtering of our data, reducing the significance of a given correlation value. This effect on reported statistics is not normally accounted for in the literature where the original number of the degrees of freedom is assumed. In contrast, the short TE correction significantly reduces correlation values, mostly in the original PCC seed region.

To interpret the accuracy of these changes in the Rest data we can consider the Rest + Motion data, as we have already shown that the short TE correction method functions across a continuum of motion artifact sizes (Fig. 3). Here we observe that the short TE correction significantly enhances the correlation values in the central and lateral nodes of the DMN, while significantly reducing correlations in superior axial planes: these results show that short TE correction is elevating DMN correlations and reducing artifactual correlations associated with large head motion. The low-pass filter does not produce as widespread or robust changes, either positive or negative (e.g., only the central PCC node of the DMN shows enhanced correlation). Extrapolating this to the Rest data, the reductions in local correlation values following short TE correction may indicate the removal of artificial correlations associated with more subtle movement artifacts.

Finally, we expect that there may be residual long-distance correlations in the Breathing data set attributed to uncorrected physiological noise. Fig. 6 highlights a slice through the ventricles, an area typically influenced by physiological noise. The enhanced correlation values in these regions following low-pass filtering suggests that residual low-frequency physiological processes (e.g., heart rate and respiratory rate variability (Birn et al., 2008a; Shmueli et al., 2007)) are amplified, resulting in false-positive DMN correlations. This problem is absent in the short TE correction method, where correlation values are significantly reduced in these areas.

### *S*_0_ and *R*_2_* coupling

Neural activity gives rise to the BOLD signal through a complex coordination of blood volume, blood flow, and metabolism changes. The classic BOLD signal increase associated with “activation” typically results from increases in blood flow and local oxygen delivery that exceed increases in oxygen metabolism. This equates to a local decrease in deoxygenated hemoglobin concentration ([dHb]) and a smaller *R*_2_*. This coupling between activation and *R*_2_* is the motivation for using multi-echo data to isolate *R*_2_* changes from concurrent *S*_0_ changes, as this quantification brings the data one degree closer to reflecting true neuronal changes.

However, the blood flow and local [dHb] changes are initiated by changes in vascular tone: the dilation or constriction of supply vessels on the arterial side results in increases or decreases of blood flow, respectively. These changes in vascular tone change the local blood volume and affect *S*_0_.

Thus, through neurovascular coupling, neuronal stimulation causes changes in both *S*_0_ and *R*_2_*. Although the dynamics of these two effects are different (Sirotin et al., 2009), they remain correlated at the timescale of typical fMRI experiments (TR ~ 2 s).

The consequences of this cross-talk are demonstrated in our data: even at a TE of 3.3 ms when BOLD signal dephasing has not had time to evolve, we observe signal fluctuations correlated with the visual stimulus, causing the short TE correction method to remove some of the BOLD activation of interest (Table 3). A previous study that explored short TE data concluded that this cross-talk reflects blood volume changes and is restricted to voxels that initially contain large blood volumes (i.e., containing large draining veins) (Buur et al., 2009). Note that study examined data acquired at a longer effective TE value (TE ~ 9 ms) than the current study.

By comparing the Visual_3.3_ and Visual_10_ data sets we are able to show that TE = 10 ms data are likely to be more confounded by this cross-talk correlation, reducing BOLD activations even further. This suggests that at TE = 10 ms we are increasing BOLD contamination in our short TE data. It is not possible to conclude whether the TE1/TE2 crosstalk reflects blood volume (*S*_0_) changes or BOLD contamination (or a combination of both) in the TE = 3.3 ms data, but our data indicate that the choice of the “short” TE results in important changes in the signal contrast, even in the range of 3.3–10 ms. Also, while data sets with small motion confounds (Visual_3.3_) show a negative impact on the BOLD activation following short TE correction, the same data with amplified head motion (Visual_3.3_ + Motion) show a positive impact: the number of significantly activated voxels in these data is actually increased by the short TE regression method. Considering the negative impact of the correction methods on the local correlation values of all data sets (Fig. 5), further exploration is needed to determine whether the short TE correction is improving or hindering the fair comparison of BOLD activation maps between data sets with differing motion artifacts, or whether it could potentially correct for blood volume contributions to the BOLD signal. For example, improvements in data acquisition to reduce the effective short TE even further would better isolate the short TE data from potential cross-talk with the BOLD signal changes of interest.

It may also be possible to interpret this “cross-talk” as a beneficial attribute: with further advances in acquisition and analysis methods, quantitative measures of cerebral blood volume using the short TE data may be obtained. This information could be incorporated into the latest models of quantitative BOLD signal changes (Bulte et al., 2012; Gauthier and Hoge, 2012) that assume a constant relationship between blood volume and blood flow, thereby making a more accurate translation between the BOLD signal and the underlying neural activation.

### Short TE correction methods

There are several methods for using short TE data to correct BOLD-weighted data, and this study focuses on the simple method of using the short TE data as voxelwise noise regressors. This assumes that the short TE data has no BOLD contamination (in effect, we assume that TE1 = 0 ms). In contrast, fitting multi-echo data for *S*_0_ and *R*_2_* does not make this assumption, as the model fit allows for all non-zero TE values to result in some level of BOLD contamination in the data. Thus, a fitting approach should yield a more accurate reflection of the BOLD signal. However, when only two TE data sets are acquired (as in the current study), the fitting method no longer has this advantage (as described in Gowland and Bowtell, 2007). Considering the existing multi-echo literature (Bianciardi et al., 2011; Gowland and Bowtell, 2007; Posse et al., 1999), acquiring additional echoes should greatly improve the ability to accurately fit for *R*_2_*. However, this would typically require longer TR values, reducing the temporal resolution of the fMRI scan, while the method presented here requires no additional scan time and could be utilized in short-TR studies.

There are several additional means by which short TE data could be incorporated into noise correction schemes. Independent component analysis (ICA) has been used to isolate noise artifacts from the remaining data of interest (Beckmann and Smith, 2004), although this typically requires the researcher to manually identify which components represent noise, which could perhaps be ambiguous and can be time consuming in large studies. Methods for automating ICA denoising have recently been proposed: multi-echo data is processed using ICA producing components that can be identified as “noise” or “signal” based on their TE-dependence (Kundu et al., 2012) or spatial similarity to patterns of known physiological processes (Perlbarg et al., 2007). With additional scan acceleration methods and new sequences, it is becoming increasingly possible to acquire several echo timepoints without dramatically lengthening scan time or lowering temporal resolution (Feng et al., 2011; Poser et al., 2006). However, without these factors, we show it is possible to achieve improved noise correction using only one additional short TE in the normally unused dead-space prior to the BOLD-weighted TE acquisition.

### Limitations of the current study

The number of subjects in this study is lower than the large numbers used in group connectivity comparison studies (i.e., 1000 subjects were included in Van Dijk et al., 2012). The impact of using short TE data to correct motion and physiological artifacts remains to be validated on larger cohorts with more typical noise properties. Also, while the variance explained by the short TE regressors is coupled with the total amount of head motion in the scan, even in cases of small head motion artifacts (Fig. 3), it is not clear how this correction method would impact the robust (default mode) versus the subtle (e.g., sub-cortical) intrinsic connectivity networks in these groups. In the small cohort of the current study, we were not able to characterize differences in more subtle connectivity networks.

We have compared TEs of 3.3 and 10 ms and shown different levels of BOLD contamination in these data. In spiral sequences, the effective TE, when the center of k-space is acquired, takes place at the beginning of the read-out, while EPI sequences generally acquire the center of k-space at the midpoint of the readout. Thus, much of our higher spatial-frequency TE1 data is acquired at times greater than 3.3 ms (or 10 ms), which may alter how we perceive BOLD contamination or correlated blood volume effects.

### Recommendations

Motion and physiological noise remain a confound in the analysis of all fMRI data, particularly in functional connectivity studies. Our results have two main implications for the field. Firstly, we have shown that physiological noise, although contributing to the data variance at short TE, is mostly corrected for using existing and accepted physiological noise regressors (Table 2, no significant difference between Breathing and Rest *F*-test results). Our results highlight the importance of collecting physiological data during all scans, including end-tidal gas measurements and the bellows and pulse oximeter data that are readily available on most MRI scanners; these data remove the gross physiological signal changes in the Breathing data without relying on global signal regression, which can create artifactual anticorrelations in the data (Murphy et al., 2009). This would enable the RETROICOR, blood gas, and cardiac/respiratory rate regressors to be modeled and removed from all functional data collected.

Secondly, we have reproduced the troubling findings of the recent literature, showing that motion artifacts (both large and small) are not sufficiently removed from BOLD fMRI data using the traditional motion transformation regressors and their derivatives. The short TE data set collected in this study had no penalty on our data acquisition: the data are collected in the unused time we typically spend waiting for BOLD contrast to evolve, thus are effectively “free” to acquire. The strong correlation between the variance explained by short TE data and the subtle head movements present in our resting state data suggests that this type of acquisition could greatly benefit a wide range of fMRI studies. To fully realize the potential of the data correction method we present, echo times would be need to be shortened even further, or multiple echoes could be collected with the assistance of acceleration techniques in order to fully isolate the noise variance from the signal variance of interest. With better hardware and pulse sequences, these changes are possible; but we have also shown that existing acquisition strategies can provide at least some benefit in correcting for the subtle motion artifacts remaining in fMRI data.

## Conclusions

We have shown that short TE fMRI data (TE = 3.3 ms) contain signal variance related to physiological fluctuations and motion artifacts. Using a dual-echo spiral acquisition, the short TE data set can be used to reduce related noise variance in the simultaneously acquired BOLD-weighted data set, with no additional “cost” in terms of scan time or temporal resolution. The extent of this variance is strongly correlated with the mean head motion across the scan across a continuum of small and large head movement artifacts. Simply regressing the voxelwise short TE data could restore some of the default mode network structure in data sets corrupted by gross head motion, reducing local artifactual correlations. This method should benefit future studies intending to compare resting connectivity between groups that exhibit differing levels of motion or noise.

The following are the supplementary data related to this article.

Table S1 The number of brain voxels in which the short TE data of the *Rest*, Rest + Motion, and Breathing data sets were significantly correlated (positive or negative correlations) with traditional noise regressors (*p* < 0.05, Bonferroni corrected). The noise regressors include six motion transformations, the derivatives of these transformations, eight RETROICOR regressors (1–4 cardiac-related and 5–8 respiratory-related), RVT convolved with the RRF, cardiac rate convolved with the CRF, end-tidal O_2_ and CO_2_ convolved with an HRF, and the mean time series of voxels in a CSF ROI drawn in the lateral ventricles. The extent of significant correlations between the short TE (3.3 ms) and BOLD-weighted TE (TE2, 35 ms) data is also provided. The group averages are shown, and bold values are significantly different in the Rest + Motion or Breathing data sets compared to the Rest data set (*p* < 0.05, paired *t*-test, corrected for multiple comparisons). Fig. 1 illustrates the correlation between the short TE data and motion regressors: subjects exhibiting the median and maximum scan motion were identified using Table 1 (subjects 5 and 3 in the Rest data and subjects 8 and 10 in the Rest + Motion data, respectively), and the motion regressor with the greatest number of correlated voxels (outlined in the above table) in that subject's data was used. Fig. 2 illustrates binary maps of significant correlation (or anticorrelation) between short TE data and the cardiac and respiratory RETROICOR regressors, as well as thresholded maps of correlations between short TE data and the RVT regressor. Subjects were selected as those exhibiting the median and maximum number of significantly correlated voxels, combining across the four cardiac and four respiratory RETROICOR regressors (outlined above).

## Figures and Tables

**Table 1:** Estimates of head motion in the Rest, Rest + Motion, and Breathing data sets. Motion was calculated as the scan average of the framewise displacement (mm).

Subject	Rest	Rest + Motion	Breathing
1	0.051	0.277	0.054
2	0.064	0.071	0.048
3	0.119	0.266	0.057
4	0.052	0.138	0.073
5	0.056	0.206	0.064
6	0.068	0.354	0.067
7	0.090	0.690	0.048
8	0.053	0.224	0.063
9	0.061	0.195	0.057
10	0.039	1.431	0.051
Average	0.065	0.385*	0.058

There was significantly more movement in the Rest + Motion data relative to the Rest data (*p = 0.035, paired *t*‐test). Subjects showing the median and maximum head motion in the Rest (subjects 5 and 3) and Rest + Motion (subjects 8 and 10) data are included in Fig. 1.

**Table 2:** Percentage of brain voxels in which the addition of short TE data regressors explains significant additional variance beyond that explained by traditional preprocessing (*F*-test, *p* < 0.05, Bonferroni corrected) and the mean *F*-statistic of these voxels in the Rest, Rest + Motion, and Breathing data sets of all 10 subjects.

	Rest				Rest + motion				Breathing			
Sub	Sig voxels	All voxels	Percent	Mean *F*-stat	Sig voxels	All voxels	Percent	Mean *F*-stat	Sig voxels	All voxels	Percent	Mean *F*-stat
1	7851	38,426	20.43	62.43	27,407	38,177	71.79	217.86	9880	38,318	25.78	67.93
2	9965	40,091	24.86	67.79	16,233	39945	40.64	105.97	9761	40,153	24.31	69.27
3	17059	41,631	40.98	97.77	34,027	41178	82.63	490.30	15,285	41,456	36.87	89.69
4	6970	32,637	21.36	57.67	21,933	33007	66.45	195.02	7943	33,528	23.69	59.32
5	11626	44,512	26.12	59.06	32196	44820	71.83	200.18	11,741	44,156	26.59	68.58
6	13265	42,651	31.10	70.46	30,012	42935	69.90	228.12	10,973	42,492	25.82	74.79
7	15810	46,319	34.13	71.47	36,445	45822	79.54	296.16	13,780	46,533	29.61	66.81
8	8901	38,711	22.99	62.54	30,427	38768	78.48	228.42	11,739	39,179	29.96	67.72
9	10647	45,016	23.65	58.12	27,659	45387	60.94	172.64	11,133	44,658	24.93	64.24
10	8977	46,756	19.20	61.28	26,946	46240	58.27	129.23	10,712	46,519	23.03	70.09
Mean			26.48	66.86			68.05*	226.39*			27.06	69.84

*The Rest + Motion data showed significantly larger percentages and higher statistics than the Rest data across the group (paired *t*‐test, *p* < 0.05) while the Breathing data were not significantly different. These results indicate the short TE data correction method is addressing motion artifacts, while physiological noise is already well modeled and removed using the traditional preprocessing regressors (e.g., RETROICOR, RVT, cardiac rate, CO_2_, and O_2_).

**Table 3:** The Visual_3.3_, Visual_10_, and Visual_3.3_ + Motion data were analyzed to obtain visual activation maps (*p* < 0.05, Bonferroni corrected) following traditional preprocessing and following the addition of short TE data regression.

Subject	TE1 = 3.3 ms			TE1 = 10 ms			TE1 = 3.3 ms (motion)		
Traditional preprocessing	TE1 regression	% Change	Traditional preprocessing	TE1 regression	% Change	Traditional preprocessing	TE1 regression	% Change
*No. of activated voxels*									
1	1103	882	− 20.0%	988	566	− 42.7%	623	1103	77.0%
2	1187	1002	− 15.6%	1366	900	− 34.1%	1738	1716	− 1.3%
4	1181	1051	− 11.0%	987	731	− 25.9%	929	1103	18.7%
5	1031	851	− 17.5%	1970	1293	− 34.4%	1116	1561	39.9%
6	1568	1354	− 13.6%	2108	1563	− 25.9%	664	1332	100.6%
8	1535	1167	− 24.0%	2646	1813	− 31.5%	1120	1413	26.2%
9	1697	1360	− 19.9%	2288	1629	− 28.8%	1053	1506	43.0%
10	2439	2193	− 10.1%	2228	1438	− 35.5%	523	684	30.8%
Average	1468	1233	− 16.5%	1823	1242	− 32.3%⁎	971	1302	41.9%⁎

*Mean t-statistic*									
1	15.2	13.5	− 11.0%	16.4	11.8	− 28.1%	14.9	18.1	21.5%
2	16.2	14.4	− 11.1%	21.9	14.9	− 31.9%	24.8	22.1	− 10.7%
4	25.4	21.7	− 14.7%	18.2	14.4	− 20.6%	19.6	21.6	10.5%
5	15.5	13.7	− 11.2%	31.9	18.2	− 43.0%	20.9	33.6	60.5%
6	20.6	16.6	− 19.5%	28.3	18.6	− 34.2%	21.9	30.4	39.1%
8	18.8	15.0	− 20.1%	35.2	19.9	− 43.4%	18.9	19.5	3.2%
9	16.0	13.7	− 14.0%	25.3	16.3	− 35.6%	13.9	21.4	53.4%
10	24.9	19.9	− 19.9%	19.4	13.2	− 32.2%	11.3	12.2	7.8%
Average:	19.1	16.1	− 15.2%	24.6	15.9	− 33.6% *	18.3	22.4	23.2% *

A mask of the occipital lobe, defined in MNI space, was aligned to the functional data, and the number of significantly activated voxels (and the mean t‐statistic of these voxels) inside this region were calculated. The use of short TE data correction in the preprocessing stream caused reduction in the extent and significance of activation in both the Visual_3.3_ and Visual_10_ data sets, and this effect was significantly larger (approximately doubled) in the Visual_10_ data. Conversely, in the Visual_3.3_ + Motion data, the short TE data correction increased the size and significance of activation, and this was also significantly different than the results of the Visual_3.3_ data.

⁎ *p* < 0.05, paired *t*‐test, corrected for multiple comparisons.
