# Supplementary material for: Removing motion and physiological artifacts from intrinsic BOLD fluctuations using short echo data
Source: Neuroimage. 2013 Jan 1;64(6):526–37. doi: 10.1016/j.neuroimage.2012.09.043 (PMC3518782; doi:10.1016/j.neuroimage.2012.09.043)
Supplement: Table S1 — The number of brain voxels in which the short TE data of the Rest, Rest + Motion, and Breathing data sets were significantly correlated (positive or negative correlations) with traditional noise regressors (p < 0.05, Bonferroni corrected). The noise regressors include six motion transformations, the derivatives of these transformations, eight RETROICOR regressors (1–4 cardiac-related and 5–8 respiratory-related), RVT convolved with the RRF, cardiac rate convolved with the CRF, end-tidal O2 and CO2 convolved with an HRF, and the mean time series of voxels in a CSF ROI drawn in the lateral ventricles. The extent of significant correlations between the short TE (3.3 ms) and BOLD-weighted TE (TE2, 35 ms) data is also provided. The group averages are shown, and bold values are significantly different in the Rest + Motion or Breathing data sets compared to the Rest data set (p < 0.05, paired t-test, corrected for multiple comparisons). Fig. 1 illustrates the correlation between the short TE data and motion regressors: subjects exhibiting the median and maximum scan motion were identified using Table 1 (subjects 5 and 3 in the Rest data and subjects 8 and 10 in the Rest + Motion data, respectively), and the motion regressor with the greatest number of correlated voxels (outlined in the above table) in that subject's data was used. Fig. 2 illustrates binary maps of significant correlation (or anticorrelation) between short TE data and the cardiac and respiratory RETROICOR regressors, as well as thresholded maps of correlations between short TE data and the RVT regressor. Subjects were selected as those exhibiting the median and maximum number of significantly correlated voxels, combining across the four cardiac and four respiratory RETROICOR regressors (outlined above). [file mmc1.doc]

Bright MG, Murphy K (*Supplementary Material)*

Removing motion and physiological artifacts from intrinsic BOLD fluctuations using short echo data


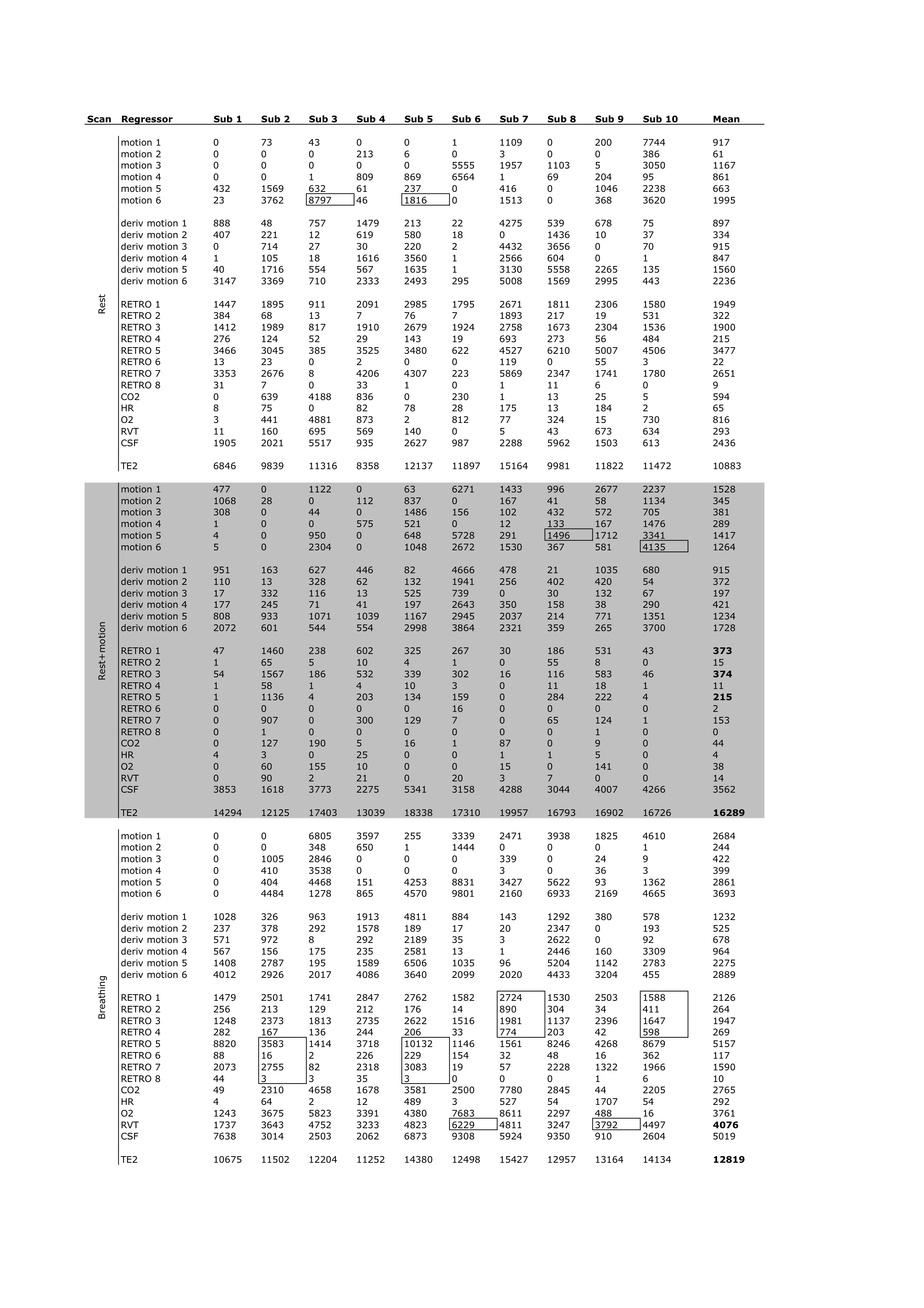


Table S1. The number of brain voxels in which the short TE data of the *Rest*, *Rest+Motion*, and *Breathing* datasets were significantly correlated (positive or negative correlations) with traditional noise regressors (p<0.05, Bonferroni corrected). The noise regressors include 6 motion transformations, the derivatives of these transformations, 8 RETROICOR regressors (1-4 cardiac-related and 5-8 respiratory-related), RVT convolved with the RRF, cardiac rate convolved with the CRF, end-tidal O_2_ and CO_2_ convolved with an HRF, and the mean timeseries of voxels in a CSF ROI drawn in the lateral ventricles. The extent of significant correlations between the short TE (3.3 ms) and BOLD-weighted TE (TE2, 35 ms) data is also provided. The group averages are shown, and bold values are significantly different in the *Rest+Motion* or *Breathing* datasets compared to the *Rest* dataset (p<0.05, paired t-test, corrected for multiple comparisons). Figure 1 illustrates the correlation between the short TE data and motion regressors: subjects exhibiting the median and maximum scan motion were identified using Table 1 (subjects 5 and 3 in the *Rest* data and subjects 8 and 10 in the *Rest+Motion* data, respectively), and the motion regressor with the greatest number of correlated voxels (outlined in the above table) in that subject’s data was used. Figure 2 illustrates binary maps of significant correlation (or anticorrelation) between short TE data and the cardiac and respiratory RETROICOR regressors, as well as thresholded maps of correlations between short TE data and the RVT regressor. Subjects were selected as those exhibiting the median and maximum number of significantly correlated voxels, combining across the four cardiac and four respiratory RETROICOR regressors (outlined above).
